# Survival prognosis evaluation in advanced breast cancer patients: a study on the application of the advanced lung cancer inflammation index

**DOI:** 10.3389/fonc.2025.1598069

**Published:** 2025-06-04

**Authors:** Chunfeng Liang, Chunyan Yang, Qiujiao Yang, Yuchen Tang, Wenhai Zhang, Qixing Tan, Qinghong Qin

**Affiliations:** Department of Breast Surgery, Guangxi Medical University Cancer Hospital, Nanning, China

**Keywords:** advanced breast cancer, survival prognosis, advanced lung cancer inflammation index (ALI), inflammation, nutritional status

## Abstract

**Background:**

Inflammation and nutritional status play critical roles in tumor initiation and progression. Advanced Lung Cancer Inflammation Index (ALI) has gained widespread attention as a novel biomarker for cancer prognosis evaluation.

**Methods:**

This retrospective study analyzed 163 advanced breast cancer patients with distant metastasis (Guangxi Medical University Cancer Hospital, 2016-2023). Patients were stratified into high-ALI (n=64) and low-ALI (n=99) groups via K-means clustering. Kaplan-Meier survival curves with log-rank testing were used to assess survival differences, while Cox proportional hazards models were employed to evaluate the independent prognostic value of ALI. The predictive performance of ALI was assessed using time-dependent ROC curves.

**Results:**

High ALI correlated with superior overall survival (log-rank p=0.0024) [HR=2.493 (95%CI 1.350-4.606) p = 0.004]. Multivariate analysis confirmed ALI as an independent prognostic factor (HR=0.39, 95% CI 0.16-0.95, p=0.037). ALI demonstrated stable predictive accuracy with 3-year AUC=0.645 and 5-year AUC=0.650 (C-index=0.65). Subgroup analyses confirmed prognostic consistency across clinical characteristics (p-interaction>0.05).

**Conclusion:**

ALI is an independent prognostic factor for advanced breast cancer patients with good predictive ability. It provides an important supplementary prognostic marker for clinical practice and can help optimize personalized treatment strategies.

## 1 Background

According to the 2022 global statistics report from GLOBOCAN (1), breast cancer ranks second in incidence and fourth in mortality among 185 countries and 36 types of cancer. In China, the incidence and mortality of breast cancer continue to rise, making it a significant public health issue (2). With the increase in medical insurance coverage and advancements in cancer screening technologies, early diagnosis of breast cancer has significantly improved, and survival rates have risen accordingly (3–5). However, some patients are diagnosed with advanced breast cancer at the initial diagnosis, with metastatic cases constituting about 38% of newly diagnosed cases (6). Treatment for advanced breast cancer requires a multidisciplinary team approach, including endocrine therapy, radiotherapy, chemotherapy, and targeted therapy (7). While precision oncology frameworks have advanced subtype-specific management, therapeutic optimization for metastatic breast cancer remains clinically challenging (8, 9). Prognostic prediction is crucial for guiding treatment strategies, but the prognosis of advanced breast cancer is difficult to predict. Therefore, new biomarkers are urgently needed to help predict the patient’s survival and enable early intervention and personalized treatment.

The growth and metastasis of tumors are closely related to systemic inflammation and nutritional status. Numerous studies have shown that nutritional and inflammatory markers can serve as effective prognostic predictors for malignant tumors (10–15). The neutrophil-to-lymphocyte ratio (NLR), a well-established inflammatory biomarker, has been widely employed in assessing treatment response, (16) monitoring complications, (17) and predicting survival in breast cancer patients. (18) NLR is calculated as the absolute neutrophil count divided by the absolute lymphocyte count in peripheral blood. Notably, elevated NLR levels are significantly associated with poorer prognosis and reduced survival rates. (19) However, NLR alone has limitations in comprehensively reflecting the complex relationship between inflammation and nutrition in cancer patients. The relationship between inflammation and cancer is inseparable because inflammation creates a favorable environment for the spread of cancer cells and triggers signaling pathways that promote carcinogenesis (20). Inflammation and the tumor microenvironment play significant roles in the progression and metastasis of breast cancer (21). Many cancer patients suffer from cachexia, and malnutrition weakens the body’s ability to fight tumors. Serum albumin (ALB), as an indicator of nutritional status, has been proven to be closely associated with poor prognosis in multiple cancer types (22–24).

The Advanced Lung Cancer Inflammation Index (ALI), originally developed for lung cancer, synergistically quantifies nutritional-inflammation equilibrium through the formula: ALI = (BMI × albumin)/NLR (25). Compared to NLR, ALI offers a more comprehensive assessment of cancer patients’ condition. By integrating NLR with BMI and albumin levels, ALI simultaneously evaluates both the inflammatory response and nutritional status, two critical determinants of cancer progression and prognosis. Although the Advanced Lung Cancer Inflammation Index (ALI) has been extensively validated for prognostic assessment in malignancies such as lung, hepatic, gastric, and colorectal cancers, robust evidence confirms that low ALI correlates with adverse oncologic outcomes (26–29). However, a critical evidence gap persists regarding its clinical utility in breast cancer, particularly among Asian populations. This discrepancy may result from aerodigestive tract malignancies altering normal anatomical structures and physiological functions, which compromises the gastrointestinal barrier, leading to malabsorption syndromes, cachexia, and systemic inflammatory responses, thereby significantly enhancing the sensitivity of ALI. Notably, advanced breast cancer induces systemic inflammatory responses and metabolic reprogramming (30–32), suggesting that the ALI may serve as a potential prognostic biomarker in this patient population.

The primary goal of this study is to explore the application of the ALI in the survival prognosis of advanced breast cancer patients, with a focus on its predictive value in Asian populations. We will analyze the significance of ALI in predicting overall survival (OS) and hope that these findings will provide new biomarkers for the prognostic evaluation of breast cancer patients, offering more effective treatment guidance for clinical practice and ultimately improving patients’ quality of life and survival duration.

## 2 Material and methods

### 2.1 Patient selection

This study, conducted through a single-center retrospective cohort analysis, aims to explore the prognostic efficacy of the ALI on overall survival (OS) in patients with advanced breast cancer. The study included 163 breast cancer patients with distant metastases confirmed during their initial hospitalization at Guangxi Medical University Affiliated Tumor Hospital (September 2016-September 2023). The inclusion criteria were as follows: (1) Pathologically confirmed primary breast cancer; (2) Distant metastasis of breast cancer confirmed through imaging studies, including positron emission tomography (PET-CT), whole-body skeletal imaging, computed tomography (CT), magnetic resonance imaging (MRI), or pathological biopsy; (3) Completion of clinical blood biomarker tests prior to surgery, radiotherapy, chemotherapy, or other treatments; (4) Complete clinical, pathological, and follow-up data. The exclusion criteria were: (1) Presence of other malignant tumors; (2) Severe underlying diseases, major organ dysfunction, autoimmune diseases, or psychiatric disorders. This study was approved by the Ethics Committee of the Guangxi Medical University Cancer Hospital (KY2023868).

### 2.2 Data collection

The survival status of the patients was followed up via telephone, and baseline data, including age, height, weight, hypertension, diabetes, marital status, education level, medical insurance, residence, and menstrual status, were collected through electronic medical records. Clinical data such as pathological type, histological grade, molecular subtype, KI-67, TNM stage, sites of distant metastasis, and treatment methods (e.g., surgery, radiotherapy, chemotherapy, etc., defined as all treatments received up to the subsequent follow-up time) were also collected. Peripheral blood data, including neutrophil count, lymphocyte count, and albumin levels, were used to calculate ALI. All baseline clinical parameters (excluding treatment regimens) were derived from the initial hematological and physical examination results obtained during patients’ first hospitalization for breast cancer - the same admission during which distant metastases were pathologically or radiologically confirmed. In this study, the follow-up period was defined as the interval between the date of first hospital admission for breast cancer (starting point) and the date of death or last follow-up (endpoint). It should be noted that due to variations in enrollment time and disease progression, not all patients had follow-up durations reaching 3 or 5 years.

### 2.3 Laboratory measurements and quality control procedures

All blood tests in this study were performed in strict accordance with the standardized protocols of Chinese public tertiary hospitals. After an 8-hour overnight fast, venous blood samples were collected from the antecubital vein in the morning and aliquoted into EDTA tubes (2 mL for complete blood count) and serum separator tubes (4 mL for liver function tests). Complete blood count analysis was performed within 2 hours after collection using a Mindray CAL8000plus automated hematology analyzer (Shenzhen, China). Liver function tests, including albumin measurement, were completed within 4 hours using a Siemens ADVIA Chemistry XPT automated biochemical analyzer (Germany). The laboratory strictly adhered to the quality control standards established by the National Center for Clinical Laboratories (NCCL), and all technicians were certified for clinical laboratory operations.

### 2.4 ALI calculation and stratification

The Advanced Lung Cancer Inflammation Index (ALI) integrates three indicators: serum albumin, body mass index (BMI), and neutrophil-lymphocyte ratio (NLR). It is calculated as: ALI = (BMI × albumin) / NLR , where:


BMI(Body Mass Index) = weight (kg) / height (m)² (unit: kg/m²)

$$NLR\left(Neutrophil-Lymphocyte\;Ratio\right)\;=\;absolute\;neutrophil\;count\;\left(\times 10^{9}/L\right)\;/\;absolute\;lymphocyte\;count\;\left(\times 10^{9}/L\right)\;\left(unitless\;ratio\right)$$

Albumin= serum albumin concentration (unit: g/L)

### 2.5 Statistical analysis

All statistical analyses were performed using R 4.3.3 and SPSS 25. Continuous variables with a normal distribution were presented as means and standard deviations, non-normally distributed continuous variables as medians and interquartile ranges, and categorical variables as percentages. Based on the distribution characteristics of ALI in the study population, K-means clustering was used to divide the patients into a high-ALI group (n=64) and a low-ALI group (n=99). Two-sample independent t-tests were used for continuous variables with normal distribution, and chi-square tests were used for categorical variables to compare baseline characteristics and incidence rates between the high-ALI and low-ALI groups. Time-to-event endpoints were analyzed using Kaplan-Meier curves with log-rank testing. To further explore the relationship between ALI and survival, Cox proportional hazards models were constructed with sequential adjustment: Model 1: Unadjusted; Model 2: Adjusted for sociodemographic covariates (age, education, insurance, hypertension, diabetes, marital status, residence, and menstrual status);Model 3: Fully adjusted for clinical-pathological variables (age, education, insurance, hypertension, diabetes, marital status, residence, menstrual status, pathological type, histological grade, and molecular subtype). Results are presented as hazard ratios (HR) with 95% confidence intervals (CI). Additionally, restricted cubic spline functions were used to visualize the dose-response relationship between ALI and OS in breast cancer. To evaluate the predictive ability of ALI at different time points (3 and 5 years), time-dependent ROC curve analysis was conducted, and the C-index was calculated. These time points were selected to align with clinical long-term prognostic assessment needs. The method accounted for censored data by utilizing all follow-up information until death or last contact, ensuring robust evaluation of ALI’s discriminative performance at predefined intervals. Finally, subgroup analysis was performed to validate the predictive ability of ALI in different populations, assessing its prognostic effect under varying patient characteristics.

## 3 Result

### 3.1 Median follow-up time

Among the 163 patients, 61 (37.4%) died. Since the Kaplan-Meier survival curve did not drop below 50% (minimum survival rate = 62.6%), the median survival time was not reached. The median follow-up was 43 months (95% CI 34.4–51.6) using reverse Kaplan-Meier estimation.

### 3.2 Baseline characteristics

K-means clustering divided ALI into high-ALI (n=64) and low-ALI (n=99) groups. Baseline characteristics were balanced between the two groups in terms of age, tumor location, tumor pathological type, molecular subtype, TNM stage, or treatment modalities(radiotherapy, chemotherapy, surgery, etc.) (all p>0.05)(**Table 1**). Specifically, regarding age distribution, the average age of patients in the high-ALI group was 52 years, while that in the low-ALI group was 50 years, with no statistically significant difference between the two groups. Concerning pathological types, whether it was invasive ductal carcinoma, invasive lobular carcinoma, or other types, the composition ratios of the two groups showed no significant differences. Among molecular subtypes, the distribution ratios of HR+/HER2-, HR+/HER2+, HR-/HER2-, and HR-/HER2+ subtypes were also comparable between the two groups. For TNM staging, the proportions of patients were basically the same in both groups. In terms of treatment modalities, the proportions of patients receiving radiotherapy, chemotherapy, and surgery were not significantly different between the two groups. This indicates that the high- ALI and low-ALI groups did not differ significantly in these confounding factors, thereby reducing their interference with survival analysis.

**Table 1:** Patient demographics and baseline characteristics.

Characteristic	ALI group		P value
	ALI<46.8, N = 99	ALI≥46.8, N = 64	
**Age(years), mean ± SD**	50 ± 11	52 ± 12	0.2572
Menstrual status, n(%)			0.3904
Postmenopausal	52 (52.5%)	38 (59.4%)	
Premenopausal	47 (47.5%)	26 (40.6%)	
Hypertension, n(%)			0.6734
No	81 (81.8%)	54 (84.4%)	
Yes	18 (18.2%)	10 (15.6%)	
Diabetes, n(%)			0.2655
No	96 (97.0%)	59 (92.2%)	
Yes	3 (3.0%)	5 (7.8%)	
Marital status, n(%)			0.9515
Married	91 (91.9%)	58 (90.6%)	
Unmarried	4 (4.0%)	2 (3.1%)	
Widow	3 (3.0%)	3 (4.7%)	
Divorced	1 (1.0%)	1 (1.6%)	
Education, n(%)			0.3904
Primary School	21 (21.2%)	12 (18.8%)	
Middle School	26 (26.3%)	17 (26.6%)	
High School	5 (5.1%)	8 (12.5%)	
Undergraduate	12 (12.1%)	10 (15.6%)	
Others	35 (35.4%)	17 (26.6%)	
Location, n(%)			0.6174
Village	55 (55.6%)	33 (51.6%)	
City	44 (44.4%)	31 (48.4%)	
Insurance, n(%)			0.2414
No	27 (27.3%)	23 (35.9%)	
Yes	72 (72.7%)	41 (64.1%)	
Pathological, n(%)			0.7915
Invasive ductal carcinoma	91 (91.9%)	61 (95.3%)	
Invasive lobular carcinoma	4 (4.0%)	2 (3.1%)	
Others	4 (4.0%)	1 (1.6%)	
Histological grade, n(%)			0.9204
2	78 (78.8%)	50 (78.1%)	
3	21 (21.2%)	14 (21.9%)	
Subtype, n(%)			0.2875
HR+/HER2-	52 (52.5%)	37 (57.8%)	
HR+/HER2+	21 (21.2%)	14 (21.9%)	
HR-/HER2-	9 (9.1%)	1 (1.6%)	
HR-/HER2+	17 (17.2%)	12 (18.8%)	
Ki 67,median(IQR)	0.35 (0.20, 0.50)	0.30 (0.20, 0.53)	0.5643
T stage, n(%)			0.3025
1	3 (3.0%)	5 (7.8%)	
2	25 (25.3%)	21 (32.8%)	
3	10 (10.1%)	6 (9.4%)	
4	61 (61.6%)	32 (50.0%)	
N stage, n(%)			0.3784
0	9 (9.1%)	6 (9.4%)	
1	36 (36.4%)	30 (46.9%)	
2	24 (24.2%)	16 (25.0%)	
3	30 (30.3%)	12 (18.8%)	
Metastasis site, n(%)			0.4454
Bone	55 (55.6%)	39 (60.9%)	
Live	20 (20.2%)	8 (12.5%)	
Lung	24 (24.2%)	17 (26.6%)	
Surgery, n(%)			0.9764
No	57 (57.6%)	37 (57.8%)	
Yes	42 (42.4%)	27 (42.2%)	
Radiation, n(%)			0.8484
No	84 (84.8%)	55 (85.9%)	
Yes	15 (15.2%)	9 (14.1%)	
Chemotherapy, n(%)			0.6844
No	31 (31.3%)	22 (34.4%)	
Yes	68 (68.7%)	42 (65.6%)	

**Figure 1:**
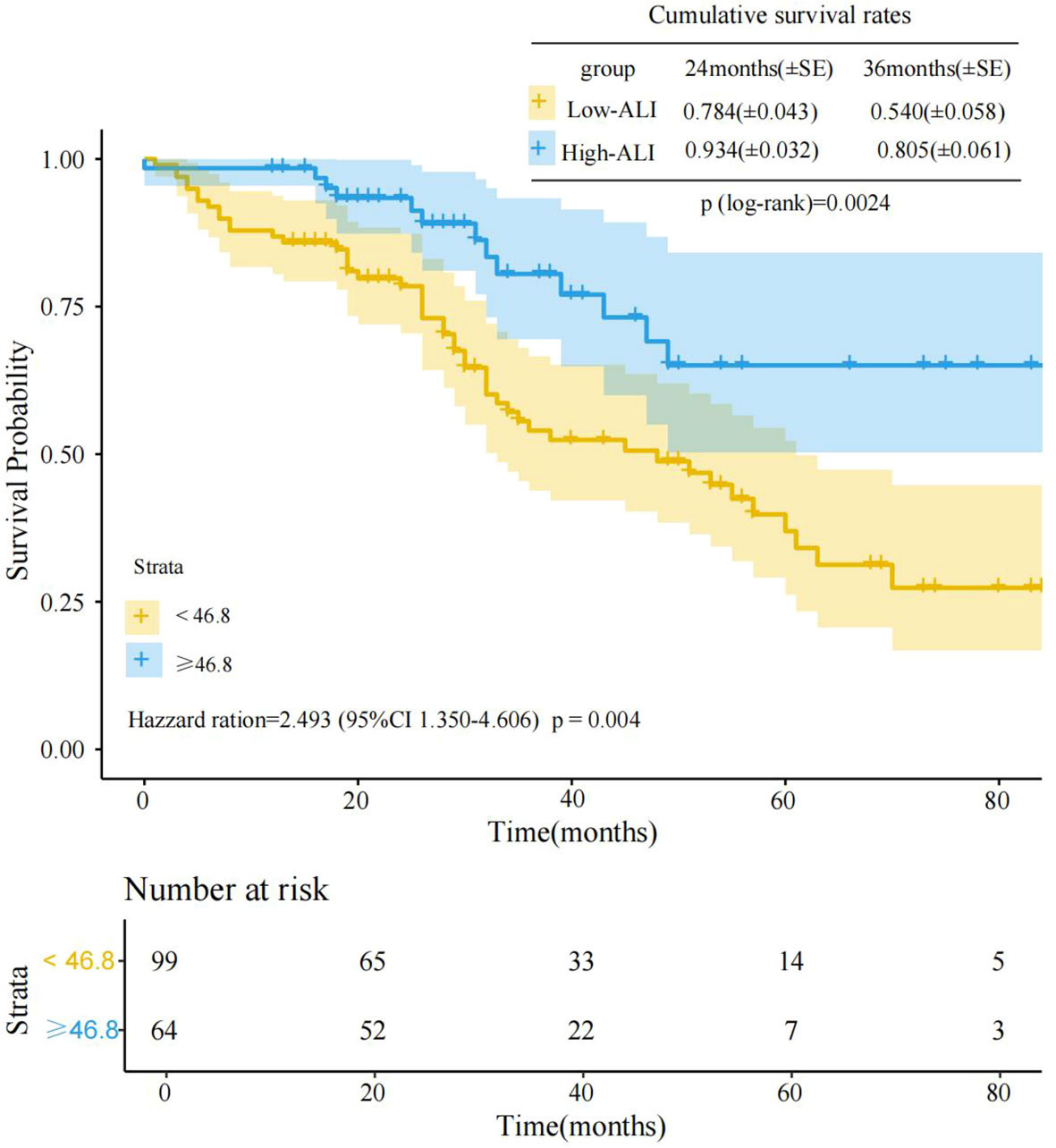
Kaplan-Meier analysis of overall survival (OS) between low-ALI and high-ALI groups.

Kaplan-Meier survival curve analysis of the two groups’ prognostic differences revealed significant survival differences between the high-ALI and low-ALI groups(log-rank p=0.0024)(**Figure 1**). As clearly shown in **Figure 1**, at the initial stage of follow-up, the survival rate curves of the two groups were relatively close. However, as time progressed, the survival rate curve of the high-ALI group was significantly higher than that of the low-ALI group. At 36 months of follow-up, the cumulative survival rate was 0.805 (80.5%) in the high-ALI group versus only 0.540 (54.0%) in the low-ALI group. Cox proportional hazards regression further quantified this association, revealing that the low-ALI group had a 2.493-fold higher risk of poor outcomes compared to the high-ALI group (HR = 2.493, 95% CI: 1.350–4.606, p = 0.004). These results robustly support ALI as an independent prognostic indicator for advanced breast cancer, with low ALI signaling significantly worse survival.

### 3.3 Dose–response relationship

**Figure 2:**
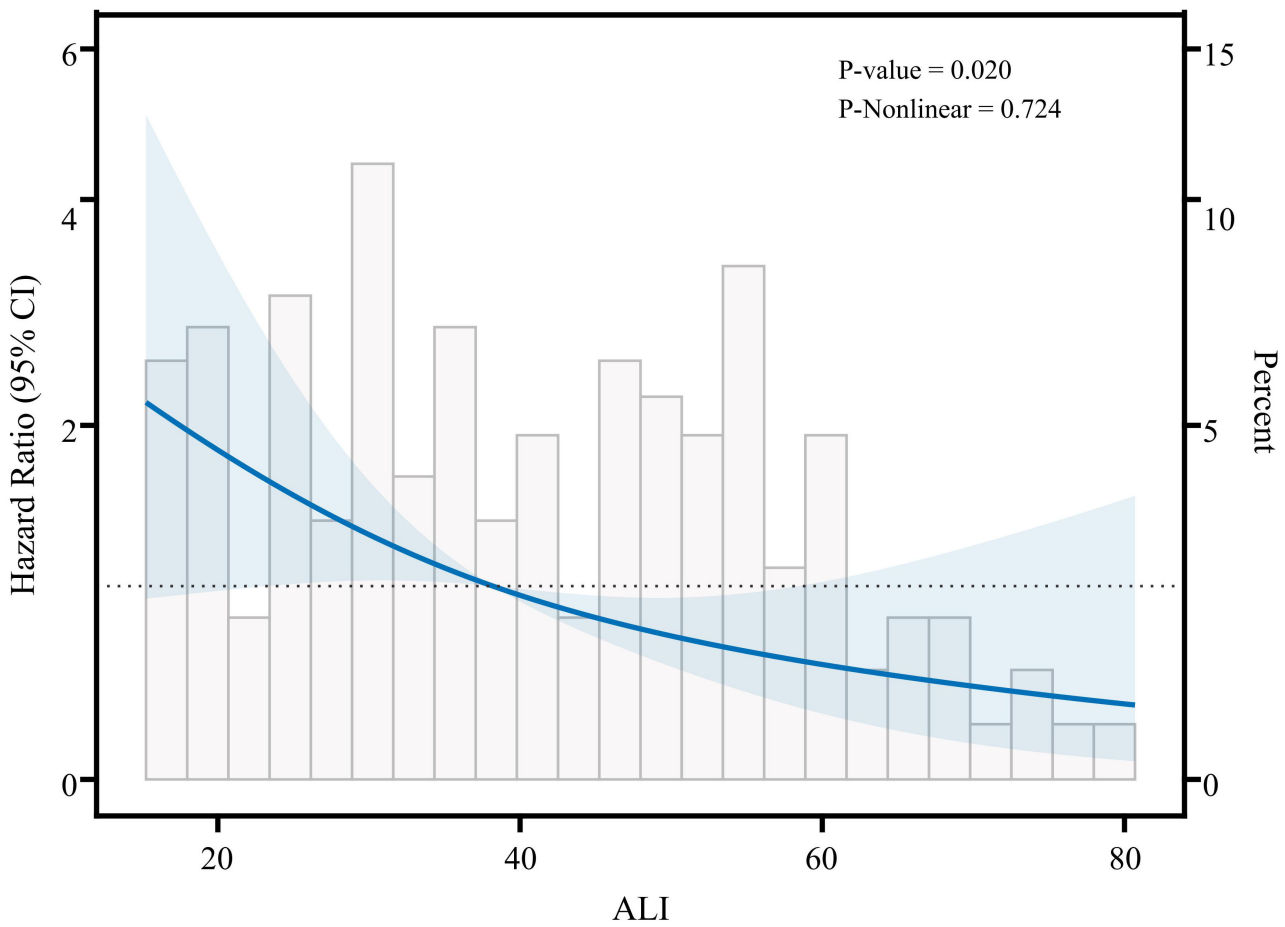
Restricted cubic spline analysis showing the relationship between ALI and survival risk in patients with advanced breast cancer.

In Cox regression models (**Table 2**), the results demonstrated a significant inverse relationship between ALI and mortality risk in advanced breast cancer patients. Three progressively adjusted models consistently revealed this protective association: When ALI was treated as a continuous variable, the unadjusted model (Model 1), the adjusted model (Model 2), and the fully adjusted model (Model 3) all suggested that the risk of death in advanced breast cancer decreased with increasing ALI; each unit increase in ALI corresponded to a 49% to 54% reduction in mortality risk. When using tertiles as a categorical variable, at least one quartile of each index was significantly related to the prognosis of advanced breast cancer, with trend tests showing statistical significance (p for trend<0.05). The three models confirmed ALI as an independent protective factor for survival in advanced breast cancer patients. In Model 2, after adjusting for clinical characteristics (e.g., age, marital status, diabetes), patients in the high-ALI group had significantly better survival compared to those in the low-ALI group (HR=0.33, 95% CI: 0.16-0.71, P=0.004). Even with further adjustments for potential prognostic factors (e.g., pathological type, molecular subtype, treatment methods) in Model 3, the prognostic significance of ALI remained (HR=0.39, 95% CI: 0.16-0.95, p=0.037). These results indicate that high ALI is an independent protective factor for survival in advanced breast cancer patients. To further explore the possible nonlinear relationship between ALI and survival, we conducted a restricted cubic spline (RCS) analysis (**Figure 2**). The results showed that no significant nonlinear association was observed between ALI and survival (p-nonlinear = 0.724). This suggests that the relationship between ALI and survival may be approximately linear, with survival time increasing linearly as ALI values rise, indicating that ALI can effectively reflect the prognosis of patients with advanced breast cancer.

**Table 2:** Association between ALI and survival (Cox regression).

Characteristic	Model- 1			Model- 2			Model- 3		
	HR	95% CI	P value	HR	95% CI	P value	HR	95% CI	P value
**ALI** (standardized)	0.51	0.34, 0.76	0.001	0.46	0.30, 0.72	<0.001	0.49	0.29, 0.82	0.007
ALI									
*Low ALI group	—	—		—	—		—	—	
Middle ALI group	0.88	0.50, 1.56	0.672	0.79	0.44, 1.42	0.428	0.8	0.39, 1.65	0.546
High ALI group	0.42	0.21, 0.83	0.012	0.33	0.16, 0.71	0.004	0.39	0.16, 0.95	0.037
**P for trend**			0.014			0.004			0.038

Model-1:no covariates were adjusted.

Model-2:adjusted for age, insurance, hypertension, diabetes, marital status, education, location, and menstrual status.

Model-3:adjusted for age, insurance, hypertension, diabetes, marital status, education, location, and menstrual status, pathological, histological grade, subtype, tumor, lymph node, metastatic, Ki 67, surgery, radiation, and chemotherapy.

*The low ALI group was set as the reference group for comparison with the medium-ALI and high-ALI groups.

### 3.4 Predictive ability of ALI

**Figure 3:**
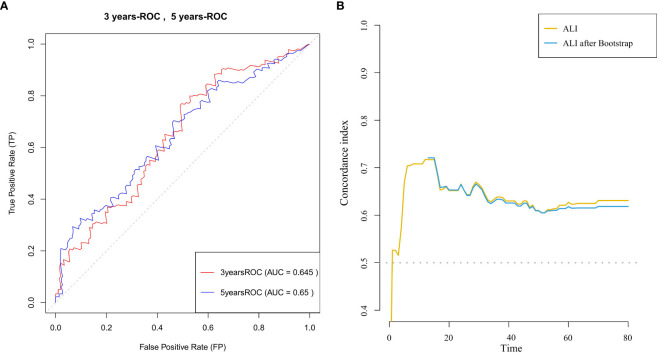
**(A)** ROC curves for 3-year and 5-year survival prediction in patients with advanced breast cancer, with the area under the curve (AUC) for each time point shown. **(B)** C-index curve illustrating the relationship between ALI and survival time in patients with advanced breast cancer.

Time-dependent ROC curve analysis **Figure 3A** was used to evaluate the predictive ability of ALI for survival in advanced breast cancer patients at different time points (3 years and 5 years). The ROC curves demonstrated consistent discriminative performance across both time points, with an AUC value of 0.645 at 3 years and 0.650 at 5 years. Time-dependent C-index analysis (**Figure 3B**) further confirmed that ALI maintained moderate predictive ability throughout the follow-up period (C-index=0.65).These results suggest that ALI is clinically valuable for predicting both short- and long-term survival in advanced breast cancer patients and can serve as an important auxiliary indicator for prognostic evaluation.

### 3.5 Stratified analysis

**Figure 4:**
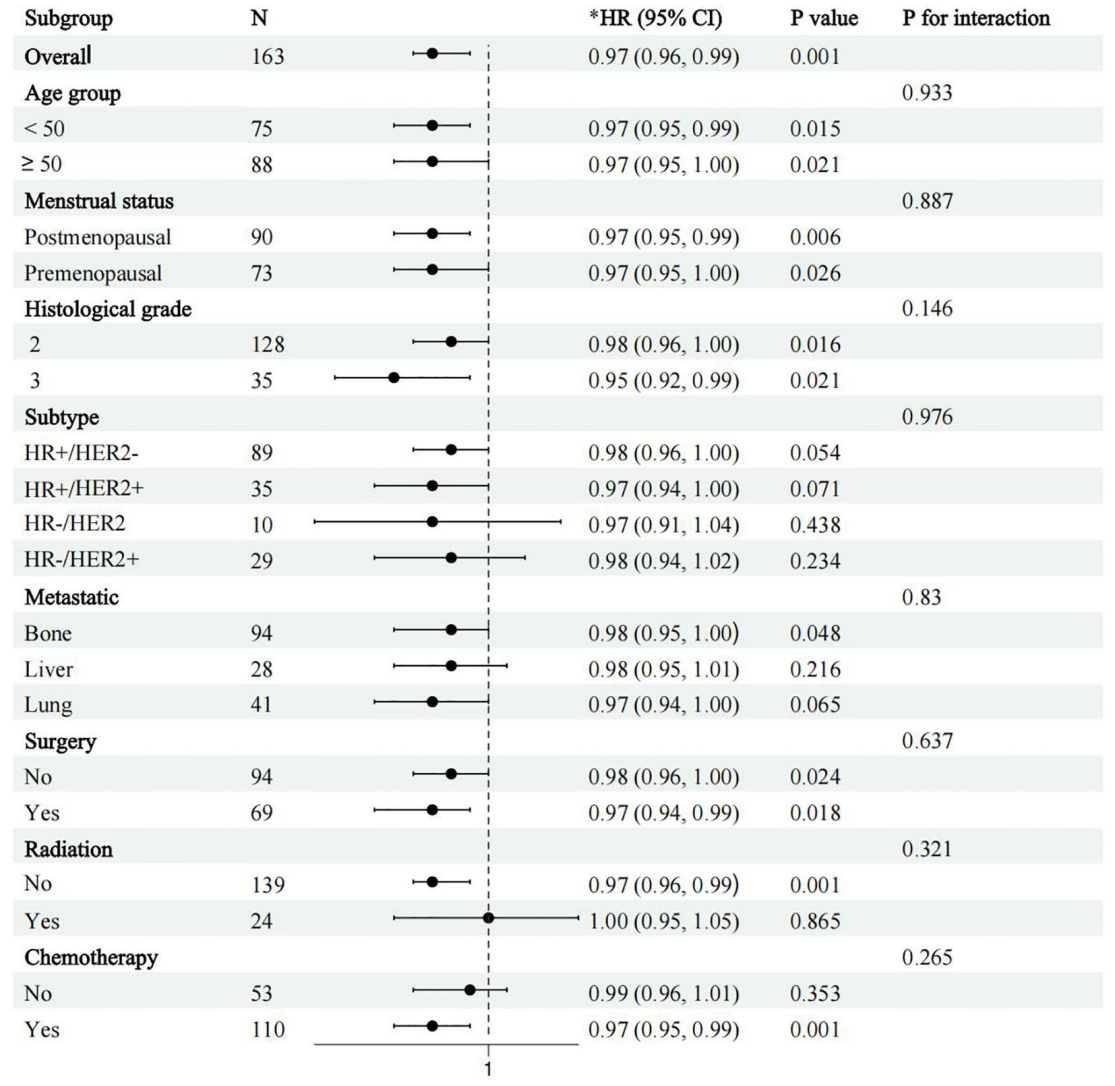
Forest plot of subgroup analysis showing survival outcomes by age, molecular subtype, and treatment modality (surgery, radiotherapy, chemotherapy, etc.) in patients with advanced breast cancer. *adjusted for age, insurance, hypertension, diabetes, marital status, education, location, and menstrual status, pathological, histological grade, subtype, tumor, lymph node, metastatic, Ki 67, surgery, radiation, and chemotherapy.

Finally, to assess the differential prognostic effect of ALI under different patient characteristics, we performed a subgroup analysis focusing on its survival prediction ability in different clinical subgroups. Patients were categorized based on age (<50 years vs ≥50 years), menopausal status (premenopausal vs postmenopausal), molecular subtype (HR+/HER2-, HR+/HER2+, HR-/HER2-, HR-/HER2+), and treatment modality (surgery, chemotherapy, radiotherapy) (**Figure 4**). Subgroup analysis showed that after adjusting for all known confounders, ALI consistently predicted survival across all subgroups (p for interaction > 0.05), indicating that ALI retains its prognostic value in various clinical contexts. This suggests that ALI has independent prognostic significance across different patient populations, unaffected by individual characteristics and treatment methods. Therefore, ALI can be considered a robust biomarker for survival prediction in advanced breast cancer patients.

## 4 Discussion

To our knowledge, this represents the first investigation applying ALI to prognostication in advanced breast cancer, extending its validation beyond gastrointestinal and lung malignancies. The results show that patients in the high-ALI group have significantly better survival compared to those in the low-ALI group. More importantly, this relationship remains independently significant in the multivariate Cox regression model (HR = 0.39). Furthermore, through 1-year and 3-year time-dependent ROC curve analysis, ALI demonstrates stable predictive ability. Although the AUC values are slightly lower, it is noteworthy that ALI, as an integrated index of inflammation and nutritional status, may compensate for the shortcomings of individual indicators, thus highlighting its potential in prognostic evaluation. Overall, ALI provides a new biomarker for survival prognosis in advanced breast cancer patients and could offer more precise personalized guidance for clinical treatment strategies.

A large number of studies have confirmed that inflammatory indices based on peripheral blood tests, such as the Systemic Immune-Inflammation Index (SII), Neutrophil-Lymphocyte Ratio (NLR), Platelet-Lymphocyte Ratio (PLR), Lymphocyte-Monocyte Ratio (LMR), C-reactive protein (CRP), and albumin (ALB),have significant value in early cancer diagnosis and screening (33), treatment monitoring (34), tumor metastasis and invasiveness (35), and prognostic evaluation (36). It is noteworthy that a meta-analysis by Mei et al. (including 66 cohort studies) showed that NLR is significantly associated with prognosis in patients with various advanced cancers, including breast cancer. High NLR values were significantly associated with poor overall survival (OS) and progression-free survival (PFS) (37). Moreover, a study conducted on an important cohort of 1763 breast cancer patients revealed that a low NLR was an independent predictor of 5-year local recurrence-free survival (LRRFS), distant metastasis-free survival (DMFS), and disease-free survival (DFS) (38). Further studies by Lafrenie et al. on triple-negative breast cancer indicated that NLR was a more accurate predictor of long-term survival than LMR. In comparison, LMR showed limited effectiveness in prognostic evaluation (39). This suggests that NLR, as a simple and readily accessible indicator, is more universal for prognostic assessment. Given the significant predictive value of NLR, this study integrates it into ALI to improve predictive performance using multi-dimensional indices.

Besides inflammatory markers, nutritional status has also been confirmed to be closely related to cancer prognosis. For example, a meta-analysis by Polański et al. demonstrated that nutritional indicators such as serum albumin, weight loss, and BMI are related to the survival and quality of life in lung cancer patients (40). A meta-analysis by Prasetiyo et al. on the impact of nutritional status on survival in breast cancer patients found that good nutritional status significantly improved survival rates (41). Although indices such as Prognostic Nutritional Index (PNI) and Controlling Nutritional Status (CONUT) mentioned in the analysis differ from ALI, they offer insights for assessing patients’ nutritional status and predicting survival prognosis. Regarding the relationship between nutritional status and tumor development, Hopkins et al. emphasized the link between obesity and cancer metabolism, discovering that a patient’s nutritional status affects metabolic pathways, alters the tumor microenvironment, and thus impacts clinical prognosis (42). Based on this evidence, ALB and BMI, which reflect energy reserves and protein metabolism status, support the integrated consideration of both factors in the prognostic evaluation of advanced breast cancer.

Currently, many studies have shown that combined indices of nutritional status and inflammatory markers perform better than single inflammatory or nutritional indicators in prognostic prediction. For example, in a prospective multicenter study, the Global Leadership Initiative on Malnutrition (GLIM) standard based on inflammatory markers had stronger predictive value for short- and long-term prognosis in cancer patients compared to the original GLIM standard (13). Similarly, in the development of cancer prognosis models, Zhu et al. demonstrated that combining an inflammation-nutrition model could significantly predict post-surgical outcomes in intrahepatic cholangiocarcinoma (43). Compared to the above studies, the innovation of ALI lies in its mathematical integration of BMI, ALB (nutrition), and NLR (inflammation), using a simplified formula to capture multi-dimensional information. This design enables ALI to combine clinical usability with predictive efficacy, especially in limited medical settings.

ALI was first applied to prognosis prediction in advanced lung cancer and has gradually been extended to other types of cancers, with low ALI levels often correlating with worse survival (44). In a study by Song M et al., which included 1,772 lung cancer patients, 16 nutritional or systemic inflammatory indices were assessed using time-ROC and C-index. Results showed that ALI had superior prognostic predictive ability compared to other inflammatory or nutritional indices for all lung cancer patients (29). As a multi-cancer prognostic biomarker, ALI has also been validated in gastric cancer, colorectal cancer, and post-surgical prognosis for hepatocellular carcinoma (45–47). Moreover, ALI is not only applicable to solid tumors but can also predict chemotherapy response and infection risks in multiple myeloma patients, with low ALI associated with a higher risk of adverse reactions (48). Consistent with previous studies, this study found that high ALI is independently associated with improved survival in advanced breast cancer (HR = 0.39), and the C-index for ALI in predicting OS at 1, 3, and 5 years in lung cancer was greater than 0.6 (29), which is consistent with findings in gastric cancer (low ALI is a risk factor for OS in gastric cancer, HR = 1.55, 95% CI: 1.11-2.16, P = 0.010) (28). Mechanistically, ALI may affect prognosis through two pathways: firstly, high NLR reflects systemic inflammation, which promotes the release of pro-inflammatory cytokines (such as IL-6, TNF-α) and drives tumor invasion via the STAT3 pathway (49, 50). Secondly, low BMI and ALB indicate a pre-cachexia state, leading to metabolic reprogramming (51, 52). These mechanisms support the findings of this study, suggesting that ALI, by integrating nutritional and inflammatory factors, serves as a potential prognostic tool for advanced breast cancer.

The detection of cytokines such as interleukin-8 (IL-8) and interleukin-17 (IL-17) holds significant importance in studying tumor progression in metastatic breast cancer patients. (53, 54) However, current detection methodologies—including enzyme-linked immunosorbent assay (ELISA), flow cytometry (FCM), and enzyme-linked immunospot assay (ELISPOT)—face substantial challenges such as high costs and technical complexity, which significantly limit their clinical translation and widespread adoption. By integrating nutritional status indicators (BMI, serum albumin) with inflammatory surrogate markers (NLR), ALI achieves reliable assessment of systemic inflammatory response. All components of this system derive from standard blood tests, featuring operational simplicity, cost-effectiveness, and strong reproducibility, enabling rapid implementation across healthcare institutions at all levels. Compared with advanced detection technologies like genomic sequencing and functional imaging, the ALI evaluation system demonstrates distinct advantages in clinical scenarios such as county-level hospitals and community-based cancer follow-up. Its “low-cost, high-efficiency” characteristics not only offer a practical tool for tumor prognosis assessment but also bridge the technological gap in systemic inflammation monitoring within primary healthcare systems.

Based on the findings of this study, the C-index for ALI remains stable over time **Figure 3B**, indicating its reliability for long-term predictive efficacy. Therefore, we recommend incorporating ALI into existing prognostic systems (e.g., TNM staging). Specifically, setting a threshold (ALI <46.8) could help identify high-risk patients, enabling more frequent follow-up or adjustments to treatment plans (e.g., early nutritional support intervention). Moreover, considering the key role of nutritional status in advanced breast cancer prognosis, we further suggest incorporating ALI into regular nutritional assessment systems, initiating multidisciplinary interventions (e.g., personalized dietary guidance, anti-inflammatory treatment) for low-ALI patients, in hopes of improving survival outcomes by correcting nutritional-inflammation imbalances.

Although previous studies have explored the relationship between the ALI and the occurrence of breast cancer (55), this study further applies this index to the survival prognosis evaluation of advanced breast cancer patients. This innovative shift not only enriches the existing literature but also provides a new perspective for clinical treatment and individualized management of advanced breast cancer. However, this study is a single-center retrospective analysis with a small sample size and single-source data. Although statistical adjustments controlled for various confounding factors, the retrospective design still limits causal inference. Nevertheless, it lays the foundation for subsequent large-sample, multi-center prospective studies.

Specifically, future research can first validate our findings. Secondly, dynamic NLR (the dynamic changes in the Neutrophil-Lymphocyte Ratio) may reflect tumor progression and immune response more sensitively than traditional static NLR. Inspired by this, Moldoveanu et al. found that dynamic NLR had better prognostic prediction in triple-negative breast cancer patients, with higher dynamic NLR changes closely associated with worse OS and PFS (56). This discovery offers new ideas for clinical treatment and prognostic evaluation in breast cancer. Future studies may explore dynamic changes in ALI to capture clinical pathophysiological changes more effectively. Additionally, although time-dependent ROC curves in this study showed stable predictive ability at 3and 5 years, the AUC values were not very high, possibly due to the high heterogeneity of breast cancer or the more significant modulation of inflammatory status by targeted treatments. Therefore, future research will further explore ALI combined with other molecular markers, immune responses, tumor microenvironment, and other potential influencing factors to refine prognostic models. Additionally, the predictive differences of ALI in different regions, ethnicities, tumor types, subtypes of breast cancer (e.g., HER2+ vs triple-negative), and different treatment strategies should be studied to provide personalized clinical references.

Notably, subgroup analysis in this study shows that the prognostic value of ALI is not affected by treatment modality (p-interaction > 0.05), suggesting that ALI may serve as a universal marker across treatment regimens. Prospective studies should clarify ALI-treatment synergies, especially for low-ALI patients who may benefit from combo therapies (e.g., nutrition + immunotherapy). Finally, we did not explore its potential molecular mechanisms or its role in assessing treatment responses in breast cancer patients receiving different therapies. These are areas that require further investigation.

## 5 Conclusion

This study confirms that ALI, as a comprehensive biomarker assessing nutritional status and inflammatory response, holds significant value in predicting the survival prognosis of advanced breast cancer patients. Moreover, the ALI system is an easy-to-use, low-cost solution for monitoring inflammation and predicting tumor outcomes, making it especially useful in areas with limited medical resources. ALI not only serves as an independent prognostic factor but also demonstrates good time-dependent predictive ability, providing a strong basis for personalized treatment in breast cancer patients. Prospective validation should evaluate dynamic ALI monitoring during treatment response and explore synergy with emerging biomarkers (e.g., ctDNA, PD-L1). Additionally, the integration of ALI with immune responses, the tumor microenvironment, and other factors should be expanded to enhance the accuracy and clinical applicability of prognostic models, promoting the realization of personalized treatment.

## Data Availability

The raw data supporting the conclusions of this article will be made available by the authors, without undue reservation.
